# Enzymatic conversion reactions of 5-hydroxymethylfurfural (HMF) to bio-based *2,5*-*diformylfuran (DFF) and* 2,5-furandicarboxylic acid (FDCA) with air: mechanisms, pathways and synthesis selectivity

**DOI:** 10.1186/s13068-020-01705-z

**Published:** 2020-04-10

**Authors:** Miša Mojca Cajnko, Uroš Novak, Miha Grilc, Blaž Likozar

**Affiliations:** Department of Catalysis and Chemical Reaction Engineering, National Institute of Chemistry, Hajdrihova 19, 1000 Ljubljana, Slovenia

**Keywords:** 5-(Hydroxymethyl)furfural (HMF), Biomass-derived furan-2, 5-Dicarboxylic acid (FDCA), Alcohol or galactose oxidase enzymes, Enzymatic reaction engineering, Air

## Abstract

**Background:**

2,5-Furandicarboxylic acid (FDCA) is one of the top biomass-derived value-added chemicals. It can be produced from fructose and other C6 sugars via formation of 5-hydroxymethilfurfural (HMF) intermediate. Most of the chemical methods for FDCA production require harsh conditions, thus as an environmentally friendly alternative, an enzymatic conversion process can be applied.

**Results:**

Commercially available horseradish peroxidase (HRP) and lignin peroxidase (LPO), alcohol (AO) and galactose oxidase (GO), catalase (CAT) and laccase (LAC) were tested against HMF, 2,5-diformylfuran (DFF), 5-hydroxymethyl-2-furoic acid (HMFA) and 5-formyl-2-furoic acid (FFA). Enzyme concentrations were determined based on the number of available active sites and reactions performed at atmospheric oxygen pressure. AO, GO, HRP and LPO were active against HMF, where LPO and HRP produced 0.6 and 0.7% of HMFA, and GO and AO produced 25.5 and 5.1% DFF, respectively. Most of the enzymes had only mild (3.2% yield or less) or no activity against DFF, HMFA and FFA, with only AO having a slightly higher activity against FFA with an FDCA yield of 11.6%. An effect of substrate concentration was measured only for AO, where 20 mM HMF resulted in 19.5% DFF and 5 mM HMF in 39.9% DFF, with a *K*_m_ value of 14 mM. Some multi-enzyme reactions were also tested and the combination of AO and CAT proved most effective in converting over 97% HMF to DFF in 72 h.

**Conclusions:**

Our study aimed at understanding the mechanism of conversion of bio-based HMF to FDCA by different selected enzymes. By understanding the reaction pathway, as well as substrate specificity and the effect of substrate concentration, we would be able to better optimize this process and obtain the best product yields in the future.

## Background

With the world’s fossil fuel resources being rapidly depleted and with an increasing concern about global warming, the production of bio-based fuels and platform chemicals from renewable sources has gained much interest [1]. Lignocellulosic biomass is an abundant and inexpensive potential source of new, greener chemicals [2]. Therefore, to switch from petroleum-based to biomass-based chemicals, new processes and technologies have to be developed [3].

**Fig. 1:**
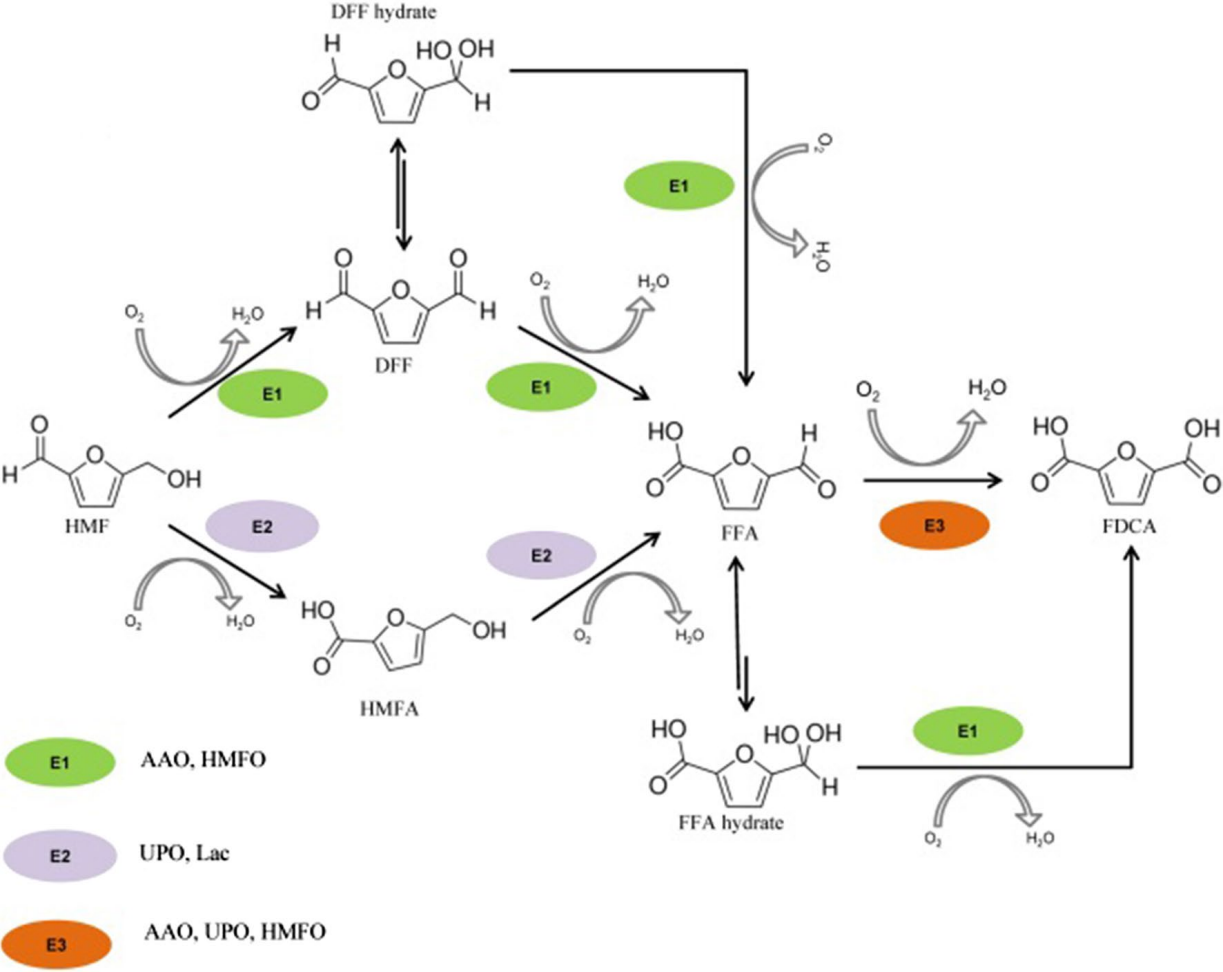
Schematic representation of oxidation routes of HMF to FDCA by different enzymes (E). Examples of enzymes for E1, E2 and E3 were taken from literature [11–14]. *AAO* aryl-alcohol oxidase, *HMFO* HMF oxidase, *UPO* unspecific fungal peroxidase, *Lac* laccase

One such important platform chemical is 2,5-furandicarboxylic acid (FDCA). FDCA and its derivates can be applied in many fields, but the most promising use of this chemical is in the replacement of tetraphthalate in oil-based plastics like polyethylene terephthalates (PET) [4, 5]. FDCA is mostly produced from fructose and other sugars via 5-hydroxymethylfurfural (HMF) as an intermediate [6]. There are two routes of HMF oxidation to FDCA (Fig. 1): (1) the aldehyde group of HMF is oxidized to form a carboxylic acid, thus yielding 5-hydroxymethyl-2-furoic acid (HMFA); then HMF acid is oxidized to yield 5-formyl-2-furoic acid (FFA) and FDCA, and (2) the alcohol group of HMF is oxidized to yield the 2,5-diformylfuran (DFF), followed by further oxidations to FFA and FDCA. Most chemical methods for production of FDCA from HMF require harsh conditions like organic solvents, high temperature and pressure and special salts. This requires high energy expenditure as well as causes environmental pollution [7–10].

An alternative to chemical synthesis of FDCA by harsh oxidation methods is biocatalysis using either whole-cell [15–19] or enzymatic [11, 13, 20] conversion processes. These are usually mild and environmentally friendly since they are carried out at lower temperatures and pressures while producing less toxic waste [21]. The whole-cell conversion process has proved to be efficient in production of FDCA [15–19] and does not require isolation of enzymes which presents an extra cost to the process. However, while these microbes produce and secrete enzymes that catalyse the oxidation of HMF to FDCA, other compounds can also be present in the reaction mixture. These are either secreted by the microbes or are a part of the growth medium for the microbe and can subsequently affect the activity of the enzyme and thus the outcome of the reaction. So in order to determine the mechanism of action of a specific enzyme, isolated enzymes have to be used.

However, only a few enzymes have been shown to be active toward HMF and/or its oxidation products, DFF, HMFA and FFA. The conversions are mostly slow and incomplete and with low yields of FDCA. Also, most of these enzymes are not capable of full conversion of HMF to FDCA and thus need to be combined in multi-enzyme reactions [11, 15, 17, 20, 22, 23]. For example, an aryl-alcohol oxidase was able to oxidase HMF to FFA with 98% yield after 4 h but only 6% FDCA after 24 h [11]. An unspecific fungal peroxygenase oxidized HMF to up to 97% HMFA after 24 h, but only 10% FDCA. 90% FDCA was obtained with oxidation of FFA after 96 h with the same enzyme [11]. A fungal laccase, with TEMPO as a mediator, was able to produce 90.2% FDCA and 8.2% FFA after 96 h [12]. The only enzyme that was capable of almost complete conversion of HMF to FDCA was HMF oxidase, an FAD-dependent oxidase that was able to oxidase HMF to 92% FFA and 8% FDCA in 5 h, and at higher enzyme concentrations to 95% FDCA after 24 h [13, 14].

Some multi-enzyme reactions were also attempted and demonstrated promising results. A combination of aryl-alcohol oxidase, galactose oxidase and unspecific peroxygenase oxidized HMF to 25% DFF and 28% HMFA after 45 min, 70% FFA after 75 min and 80% FDCA after 24 h [20]. A galactose oxidase GOaseM3-5 and an aldehyde oxidase PaoABC produced 97% FDCA after 1 h [24] and a combination of PaoABC, galactose oxidase, catalase and horseradish peroxidase produced 100% FDCA after 6 h [23].

As can be seen from previous research, some fast and complete conversions have already been achieved and an insight into the mechanism of action of these selected enzymes has been provided. However, not all enzymes work the same way, even if they belong to the same group [20, 22]. Thus, it is evident that in order to determine the most efficient enzyme or enzyme combinations for production of FDCA, understanding the mechanism of action for each individual enzyme is crucial. In our current work, we selected six different commercially available enzymes: alcohol oxidase, galactose oxidase, horseradish peroxidase, laccase, catalase and lignin peroxidase. Although HMF can be produced from fructose and other sugars [6], we used a commercially available analytical standard grade HMF to avoid any possible contaminants that could arise from its isolation from biomass. We determined their substrate specificity as well as the HMF oxidation route they employ and the effect of substrate concentration and/or cofactor on enzyme activity. Oxygen content in solution was measured during the reaction to determine the need for its supplementation. Finally, we designed some simple one-pot multi-enzyme reactions with the aim of increasing the productivity of selected enzymes. Some of the enzymes are composed of multiple units (multiple active sites), so in order to obtain and compare the activity per one active site, the enzyme concentrations were calculated based on the number of subunits of a specific enzyme. The aim of our study was not to improve the conversion process of HMF to FDCA, as that has already been achieved in previous studies, but to better understand the mechanism of conversion of HMF to FDCA by our selected enzymes: substrate selectivity, HMF oxidation route, effect of substrate concentration and effect of cofactor concentration.

## Results and discussion

### Oxidation of HMF with alcohol oxidase

**Fig. 2:**
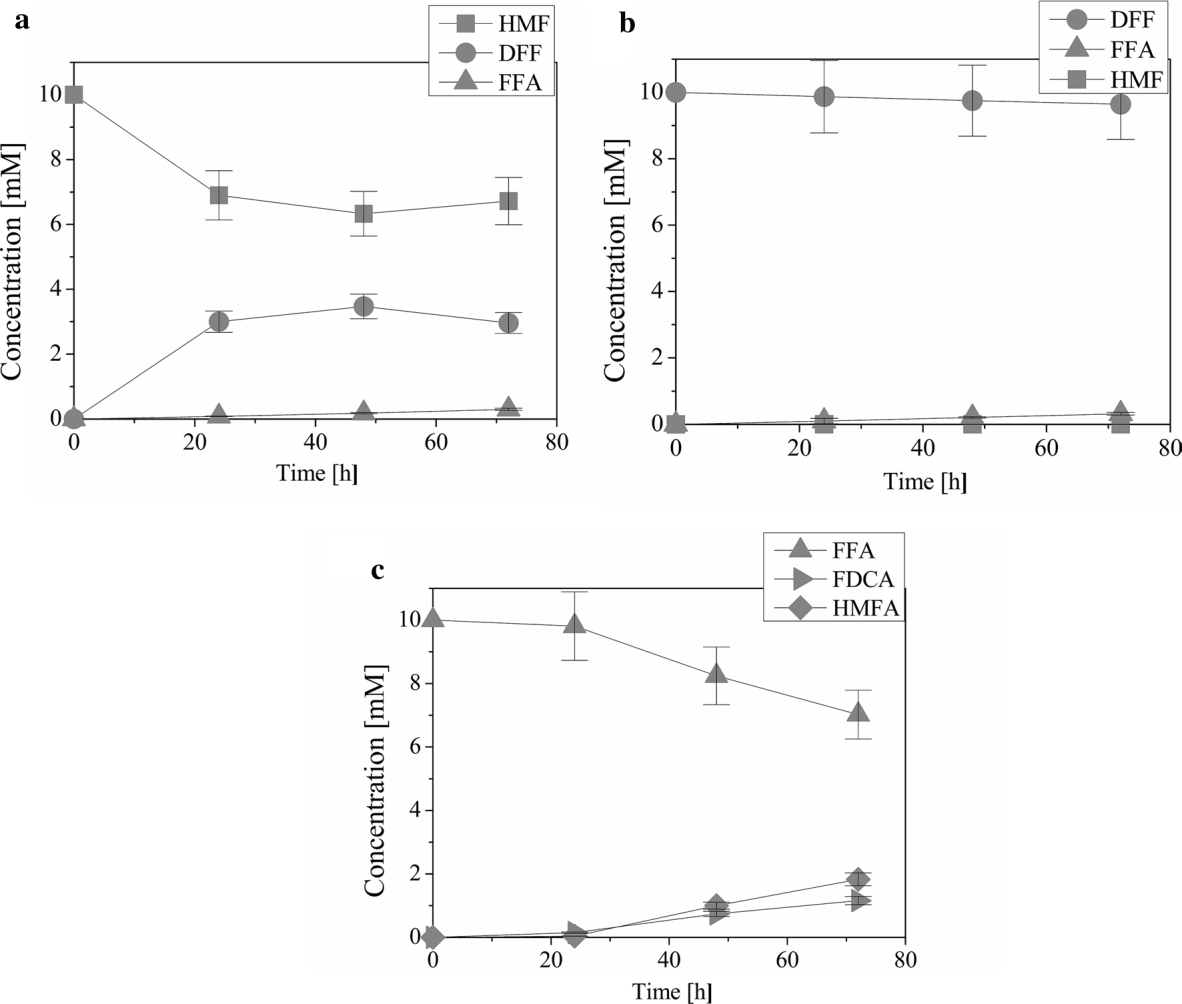
Oxidation of different substrates with AO and FAD as a cofactor. Time course of oxidation of HMF (**a**), DFF (**b**) or FFA (**c**) by AO. Reaction conditions: final reaction volume 5 mL, 1 µM enzyme, 1 µM FAD, 10 mM HMF in 50 mM sodium phosphate buffer (pH 7) at 30 °C and constant stirring at 150 min^−1^. *HMF* 5-hydroxymethilfurfural, *DFF* 2,5-diformylfuran, *FFA* 5-formyl-2-furoic acid, *FDCA* 2,5-furandicarboxylic acid, *AO* alcohol oxidase from *Pichia pastoris*, *FAD* flavin adenine dinucleotide. The average relative error was ± 11% and was estimated based on selected repeated experiments

Alcohol oxidase (AO) with 1 µM flavin adenine dinucleotide (FAD) was first tested against 10 mM HMF. Samples were taken after 24, 48 and 72 h. The results in Fig. 2a show a steep rise in DFF content after 24 h with some FFA also being formed. There was an additional increase of DFF after 48 h and a slight decrease after 72 h. The FFA content slowly increased throughout the whole incubation period, but did not reach more than a few percent.

During AO substrate oxidation H_2_O_2_ is formed which has an inhibitory effect on the enzyme [24]. Thus, the decrease in conversion of HMF to DFF after 48 h can be explained by decreased enzyme activity due to H_2_O_2_ accumulation. A small part of the decrease in DFF content could also be assigned to its conversion to FFA.

Previous research has shown that H_2_O_2_ produced by the action of an alcohol oxidase can also oxidase HMF and/or its oxidation products [11]. Therefore, we incubated HMF with H_2_O_2_ and analysed the oxidation products of the reaction after 72 h. There was only minimal (around 0.1%) conversion of HMF to DFF (data not shown), and thus, we could conclude that the oxidation of HMF was executed by the enzyme and not H_2_O_2_.

According to literature, the enzymes use molecular oxygen as a terminal electron acceptor [13, 24]. To ensure that the oxygen concentration in solution remains stable or does not drop too low to affect the activity of the enzymes, Quin et al. [22] used daily air bubbling. We measured oxygen concentration at the beginning of the experiment and then every 24 h. Results in Table 1 show that despite the enzyme activity, which was presented as conversion of HMF to DFF, the oxygen concentration remained stable during the whole process.

**Table 1:** Measurements of oxygen content in the reaction solution

Time [h]	0	24	48	72
O_2_ [mg mL^−1^]	2.80 ± 0.08	2.58 ± 0.03	2.59 ± 0.08	2.6 ± 0.1

**Fig. 3:**
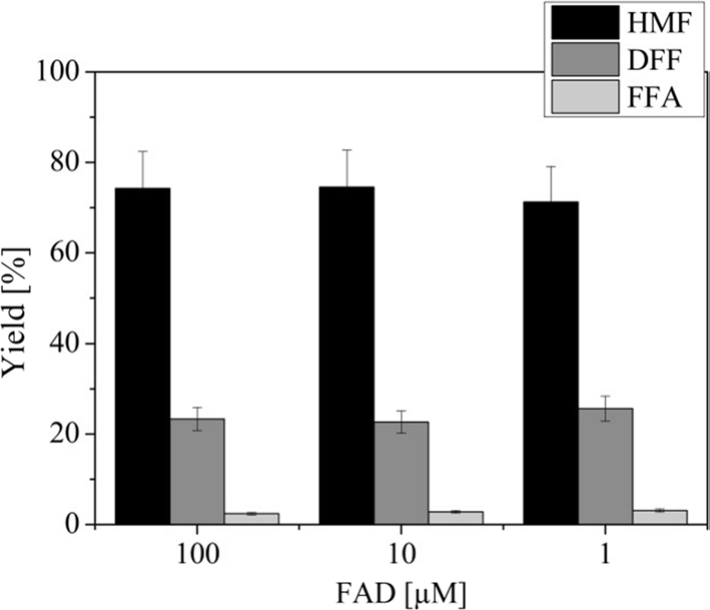
The effect of FAD concentration on activity of AO against HMF. Oxidation of HMF with AO and different concentrations of FAD after 72 h. Reaction conditions: final reaction volume 5 mL, 1 µM enzyme, 1, 10 or 100 µM FAD, 10 mM HMF in 50 mM sodium phosphate buffer (pH 7) at 30 °C and constant stirring at 150 min^−1^. *HMF* 5-hydroxymethilfurfural, *DFF* 2,5-diformylfuran, *FFA* 5-formyl-2-furoic acid, *AO* alcohol oxidase from *Pichia pastoris*, *FAD* flavin adenine dinucleotide. The average relative error was ± 11% and was estimated based on selected repeated experiments

AO is an FAD-dependent enzyme and previous research has shown that FAD concentration can have a notable effect on the activity of this type of enzyme [13]. There they determined that the best enzyme-to-cofactor molar ratio was 1:1 and a tenfold increase in FAD had a notable inhibitory effect. Therefore, we used different FAD concentrations with AO and tested them against HMF. The results in Fig. 3 show that in the case of AO, the concentration of FAD does not have an effect on the activity of the enzyme. Thus, all of the following experiments with AO were carried out with 1 µM FAD.

### Enzymatic conversion of HMF, DFF, HMFA and FFA by other enzymes

To determine substrate specificity and productivity, as well as which HMF oxidation route was employed, we tested each enzyme against HMF and its oxidation products, DFF, HMFA and FFA. Enzyme concentrations used were based on the number of their subunits and thus the measured activity was the activity of one enzyme active site. The products and product yields after 72 h of incubation are presented in Table 2 and the time course of oxidation of HMF, DFF and FFA with AO is presented in Fig. 2a–c (oxidation of HMFA is not included as no product was formed). The highest activity against HMF was measured for AO where 25.5% of DFF and 3.1% of FFA was formed. The second highest activity was measured for GAO, but with only 5.1% of DFF and no FFA. LPO and HRP showed some low activity, but instead of DFF, HMFA was formed. CAT and LAC were not active against HMF. The activity of all enzymes against DFF was low, with the highest of 3.2% of FFA being obtained with AO. The reaction was also reversible and some HMF was formed. GAO, LAC, LPO and HRP showed some activity against HMFA forming FFA and FDCA. The highest yield of FDCA was 4% and was obtained with HRP. All of the enzymes showed activity against FFA. All but one produced only small amounts of FDCA, between 0.6 and 3.2%, whereas AO produced 11.6%. The reaction was reversible in some cases. CAT, LAC and LPO produced only small amounts of HMFA and AO produced a relatively large amount of 18.2%. There was also some spontaneous conversion of substrates in the absence of an enzyme. HMF converted to 0.3% DFF, DFF to 1% FFA and 0.5% HMF and FFA to 0.5% FDCA. This spontaneous conversion shows that the already low activity of some enzymes is in fact even lower or non-existent.

**Table 2:** Oxidation of HMF, DFF, HMFA and FFA with different enzymes after 72 h

Yield [%]	Enzyme						No enzyme
	AO	GAO	CAT	LAC	LPO	HRP	
Substrate: HMF							
HMF	71.2	94.9	100	100	99.4	99.3	99.7
DFF	25.6	5.1	–	–	–	–	0.3
HMFA	–	–	–	–	0.6	0.7	–
FFA	3.1	–	–	–	–	–	–
FDCA	–	–	–	–	–	–	–
Substrate: DFF							
HMF	0.4	2.1	0.7	0.7	0.7	0.7	0.5
DFF	96.4	96.6	98.2	98.0	98.2	98.4	98.5
HMFA	–	–	–	–	–	–	–
FFA	3.2	1.3	1.1	1.3	1.1	0.9	1
FDCA	–	–	–	–	–	–	–
Substrate: HMFA							
HMF	–	–	–	–	–	–	100
DFF	–	–	–	–	–	–	–
HMFA	100	97.1	100	99.9	96.4	95.4	–
FFA	–	2.7	–	–	0.4	0.6	–
FDCA	–	0.2	–	0.1	3.1	4.0	–
Substrate: FFA							
HMF	–	–	–	–	–	–	–
DFF	–	–	–	–	–	–	–
HMFA	18.2	–	0.3	0.2	0.9	–	–
FFA	70.2	99.4	99.1	98.6	95.9	99.3	99.5
FDCA	11.6	0.6	0.6	1.1	3.2	0.7	0.5

Reaction conditions: final reaction volume 5 mL, 1, 2 or 8 µM enzyme (1 µM AO, 2 µM CAT and 8 µM GAO, LAC, LPO and HRP) or no enzyme (control), 10 mM HMF, DFF, HMFA or FFA in 50 mM sodium phosphate buffer (pH 7) at 30 °C and constant stirring at 150 min^−1^. Reactions with AO also included 1 µM FAD. *HMF* 5-hydroxymethilfurfural, *HMFA* 5-hydroxymethyl-2-furoic acid, *DFF* 2,5-diformylfuran, *FFA* 5-formyl-2-furoic acid, *FDCA* 2,5-furandicarboxylic acid, *AO* alcohol oxidase from *Pichia pastoris*, *GAO* galactose oxidase from *Dactylium dendroides*, *CAT* catalase *Aspergillus niger*, *LAC* laccase from *Trametes versicolor*, *LPO* fungal lignin peroxidase, *HRP* horseradish peroxidase. The average relative error was ± 11% and was estimated based on selected repeated experiments

In previous research only a few enzymes have been tested against HMF and even in those cases many were not tested against other oxidation products (DFF, HMFA and FFA) and/or they were tested in combination with others in multi-enzyme reactions. A similar enzyme to our AO, a fungal aryl-alcohol oxidase (AAO), was tested against HMF, DFF, HMFA and FFA. HMF and DFF both presented as good substrates for this enzyme yielding 90 or more percent of FFA after 4 h and also some FDCA after 24 h [11]. In our case, only 25.6% DFF, 3.1% FFA and no FDCA was produced after 72 h. DFF was shown to be a good substrate for AAO, because it readily forms a hydrate at neutral pH [11, 25]. However, that has proven not to be the case with our AO since only low quantities of FFA and no FDCA have been formed. As in the case of AAO [11], AO also showed no activity against HMFA. Because FFA does not readily form a hydrate, AAO was not active against this substrate [11], however, our AO was. On the other hand, another study showed that certain AAOs did have some activity against FFA [20] which was similar to our AO.

A recombinant galactose oxidase was previously tested against HMF and produced DFF with no FFA. This GAO was also capable of oxidizing HMFA, but not DFF, to FFA [20]. The oxidation of HMF with our GAO also produced only DFF, however, it was also active against DFF, producing some FFA, and against HMFA, producing not only FFA, but also small amounts of FDCA. Another group used the same GAO used in our study and tested it against HMF with similar results, 2% of DFF after 72 h. Like AO, GAO also produces H_2_O_2_ as a by-product which inhibits its action [22], thus removing this by-product might increase the enzymes activity.

To our knowledge, only one group tested CAT and HRP in a single-enzyme reaction and even then they were only tested against FFA where they produced around 55% or 22% FDCA after 48 h [20]. CAT and HRP were previously also tested in tandem against HMF, but the reaction yielded no products [22]. Both of these enzymes were mainly used in multi-enzyme reactions with AAO, AO or GAO for removal of the accumulated H_2_O_2_ [22, 23].

In previous research laccases have been used either in free form [22] or immobilized [12]. In both cases, TEMPO was used as a mediator. In the case of free enzyme reactions they tested three different LACs against HMF and obtained DFF, FFA and FDCA in yields of 68–82%, 4–6% and 5–10% after 48 to 96 h, respectively [22]. In the case of immobilized enzyme, the conversion of HMF to FDCA was over 90% in 96 h, showing a stabilizing effect of immobilization on the enzyme. Our results show a very low activity of this enzyme against DFF, HMFA and FFA and no activity against HMF, thus, emphasizing the need for a mediator.

LPO was chosen for our research because, like the unspecific peroxygenases (UPOs) used in previous research [11, 20], it is a heme-peroxidase [26]. These heme-peroxidases have been shown to catalyse a H_2_O_2_-dependent hydroxylation of alcohols into aldehydes and carboxylic acids [27, 28]. Therefore, like in the case of CAT and HRP, UPO was also used in multi-enzyme reactions for removal of the produced H_2_O_2_ [11, 20]. When HMF was oxidized by UPO, HMFA instead of DFF was formed with a yield of 72% after 72 h [11]. Our results with LPO also show this enzyme employs the HMFA oxidation route of HMF, however, the yields were much lower. Also, the reaction with UPO continued on to form FFA and FDCA, whereas the reaction with LPO did not. Despite UPO being a H_2_O_2_-dependent enzyme, previous research has also shown that it is active even in the absence of H_2_O_2_ and that the addition of this cofactor only mildly increases the production of FDCA from FFA. The increase of FDCA production seemed to be due to FFA oxidation with H_2_O_2_, which was further supported by results that showed that adding CAT, which degrades H_2_O_2_, also decreased the production of FDCA [20]. Thus, our reactions with LPO were performed in the absence of exogenous H_2_O_2_ and showed mild activity against all of the tested substrates.

The differences in our results compared to those presented in literature can not only be explained by differences in enzymes used, but also in different enzyme and substrate concentrations and their ratios as well as reaction conditions. It has been shown that the same types of enzymes from different origins have different activities and different pH optimums [20, 22]. The change in pH of the reaction solution can not only affect the activity of the enzyme, but also the HMF oxidation route it employs (DFF or HMFA) [20]. Also, some of the enzymes used in these studies are composed of multiple units (AO—octamer, CAT—tetramer) and thus have multiple active sites with which to oxidize a substrate. To our knowledge, previous research not only compared different enzymes at different concentrations, but it also did not take into account the different number of active sites on each enzyme. In our current study, we determined the enzyme concentrations based on the number of subunits (active sites) which enabled us to compare enzyme activities based on a single active site.

### Effect of substrate concentration on enzyme activity

To determine if the substrate concentration affects enzymatic activity, we tested the enzymes against 20, 10 or 5 mM of selected substrate—one enzyme against one substrate. Results in Table 3 show that substrate concentration had the most notable effect in the case of AO and HMF. At 20 mM HMF, the conversion to DFF and FFA was 19.8% and 3.7%, respectively. At lower substrate concentrations the conversion to FFA decreased to 3%; however, the production of DFF markedly increased and reached almost 40% in the case of 5 mM HMF. In the case of other enzymes, the conversion rates remained similar and thus showed no effect of substrate concentration on enzyme activity.

**Table 3:** Effect of substrate concentration on enzyme activity

Yield [%]	Substrate concentration [mM]					
	20	10	5	20	10	5
	AO + HMF			GAO + DFF		
HMF	76.5	67.2	57.1	2.3	2.2	2.1
DFF	19.8	29.7	39.9	96.4	96.5	96.6
HMFA	–	–	–	–	–	–
FFA	3.7	3.1	3.0	1.3	1.3	1.3
FDCA	–	–	–	–	–	–
	LAC + HMFA			HRP + FFA		
HMF	–	–	–	–	–	–
DFF	–	–	–	–	–	–
HMFA	100	99.9	100	–	–	–
FFA	–	–	–	99.1	99.4	99.3
FDCA	–	0.1	–	0.9	0.6	0.7

Oxidation of different concentrations of HMF, DFF, HMFA and FFA with different enzymes after 72 h. Reaction conditions: final reaction volume 5 mL, 1 or 8 µM enzyme (1 µM AO and 8 µM GAO, LAC and HRP), 5, 10 or 20 mM HMF, DFF, HMFA or FFA in 50 mM sodium phosphate buffer (pH 7) at 30 °C and constant stirring at 150 min^−1^. Reactions with AO also included 1 µM FAD. *HMF* 5-hydroxymethilfurfural, *HMFA* 5-hydroxymethyl-2-furoic acid, *DFF* 2,5-diformylfuran, *FFA* 5-formyl-2-furoic acid, *AO* alcohol oxidase from *Pichia pastoris*, *GAO* galactose oxidase from *Dactylium dendroides*, *CAT* catalase from *Aspergillus niger*, *LAC* laccase, *HRP* horseradish peroxidase. The average relative error was ± 11% and was estimated based on selected repeated experiments

The effect of substrate concentration on enzymatic activity was previously tested on a two-enzyme reaction with an aldehyde oxidase PaoABC and a galactose oxidase GOase M3-5 [24]. At 10 mM HMF, an almost complete conversion to FDCA took place, with DFF and FFA intermediates being formed. By increasing the concentration of HMF to 20 mM, the production of FDCA was reduced by half along with formation of 50% of HMFA. Another multi-enzyme reaction, a combination of galactose oxidase, HRP and PaoABC, was also tested [23]. There they showed a similar trend where high HMF concentrations resulted in a reduced production of FDCA and an increased production of HMFA. This was countered by reducing the concentration of PaoABC which resulted in a complete conversion to FDCA without any HMFA being formed. This goes to show that not only pH [20], but also substrate and enzyme concentrations can affect conversion rates and the oxidation route of certain enzymes.

Based on the reactions presented in Table 3, approximate kinetic parameters were also calculated. The time course oxidation of HMF with AO (Fig. 2a) showed that most of the substrate was converted in the first 24 h, therefore the kinetic parameters were determined based on the amount of product formed in that time period. The results in Table 4 show that the highest *V*_0_ were calculated for AO with HMF, ranging from 0.08 to 0.2 mM/h, and the lowest for LAC with HMFA, with the highest being 0.0002 mM/h and the rest at 0 mM/h. AO also had the highest affinity for its substrate, with a *K*_m_ of 14 mM. However, the highest *V*_max_ was determined for GAO with DFF, despite the *K*_m_ of 770 mM showing a very low affinity for this substrate. Also, the productivity of HRP with FFA (product yield: 0.6–0.9%) was lower than that of GAO with DFF (product yield: 1.3%), yet the *K*_m_ value of HRP with FFA was calculated to be 260 mM, almost 3 times lower than that of GAO with DFF. This discrepancy could be due to: (1) the fact that we only tested 3 different substrate concentrations (enzyme saturation was not reached); (2) that the conversion yields of DFF and FFA were very low and thus less accurate, and (3) that the Lineweaver–Burk plot is prone to error [29]. Thus, the values of *V*_max_ and *K*_m_ are most likely an overestimation.

**Table 4:** Kinetic parameters for oxidation of different concentrations of HMF, DFF, HMFA and FFA with AO, GAO, LAC and HRP after 24 h

Enzyme	Substrate	Substrate concentration [mM]	*V*_0_ [mM/h]	*V*_max_ [mM/h]	*K*_m_ [mM]
AO	HMF	5	0.08	0.3	14
		10	0.1		
		20	0.2		
GAO	DFF	5	0.006	1	770
		10	0.01		
		20	0.02		
LAC	HMFA	5	0.0000	nd	nd
		10	0.0000		
		20	0.0002		
HRP	FFA	5	0.001	0.07	260
		10	0.002		
		20	0.004		

Reaction conditions: final reaction volume 5 mL, 1 or 8 µM enzyme (1 µM AO and 8 µM GAO, LAC and HRP), 5, 10 or 20 mM HMF, DFF, HMFA or FFA in 50 mM sodium phosphate buffer (pH 7) at 30 °C and constant stirring at 150 min^−1^. Reactions with AO also included 1 µM FAD. *HMF* 5-hydroxymethilfurfural, *HMFA* 5-hydroxymethyl-2-furoic acid, *DFF* 2,5-diformylfuran, *FFA* 5-formyl-2-furoic acid, *AO* alcohol oxidase from *Pichia pastoris*, *GAO* galactose oxidase from *Dactylium dendroides*, *CAT* catalase from *Aspergillus niger*, *LAC* laccase, *HRP* horseradish peroxidase. The average relative error for raw data was ± 11% and was estimated based on selected repeated experiments

To the best of our knowledge, kinetic parameters for this reaction have been determined for only a few enzymes [11, 13, 14, 20]. The highest affinities presented in literature were determined for UPO and DFF with *K*_m_ of 0.8 mM [20] and HMFO and HMF with *K*_m_ of 1.4 mM [13]. A *K*_m_ of 1.6 mM was determined for one AAO with HMF [11], which is almost 10 times lower than that of our AO. However, in another study, two other AAOs were tested against HMF and their *K*_m_ values were 36.3 and 7.2 mM, respectively [20]. The same group also tested GAO with HMF and UPO with HMF and DFF. The *K*_m_ value for GAO with HMF was 142 mM, indicating a relatively low affinity for this substrate. The group did not test GAO with DFF and, thus a comparison to our results is not possible. Although our calculated *K*_m_ value for GAO with DFF is most likely an overestimation, based on the data on GAO with HMF [20] and the fact that our results showed the conversion of DFF with GAO is lower than that of HMF, a high value of *K*_m_ for DFF was to be expected. Also to be taken into account is the type of measurement used to obtain the raw data for the determination of kinetic parameters. In our study, we directly measured the amount of product formed, whereas some other groups used a more indirect method and measured the amount of H_2_O_2_ produced during the enzymatic conversion of a substrate in a peroxidase-coupled assay [11, 13, 14]. Since different measurements yield different types of data, a comparison of our results to those presented in literature might not be very accurate.

### One-pot multi-enzyme reactions

**Fig. 4:**
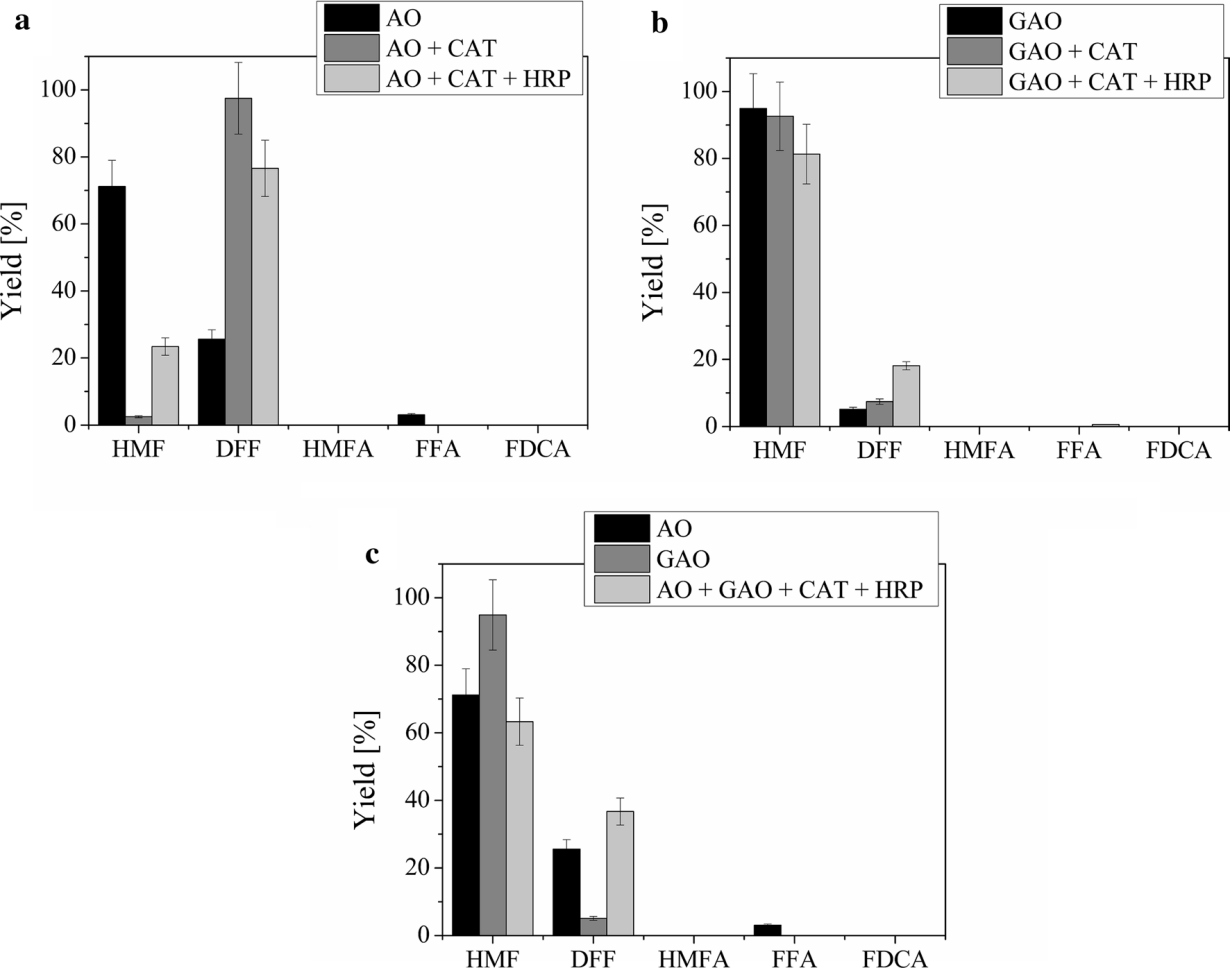
One-pot multi-enzyme reactions. Oxidation products of HMF with one or more enzymes after 72 h. **a** AO alone or with CAT or CAT and HRP. Reaction conditions: final reaction volume 5 mL, 1 µM AO and 2 µM CAT or 1 µM AO, 2 µM CAT and 8 µM HRP, 1 µM FAD, 10 mM HMF in 50 mM sodium phosphate buffer (pH 7) at 30 °C and constant stirring at 150 min^−1^. **b** GAO alone or with CAT or CAT and HRP. Reaction conditions: final reaction volume 5 mL, 8 µM GAO and 2 µM CAT or 1 µM AO, 2 µM CAT and 8 µM HRP, 10 mM HMF in 50 mM sodium phosphate buffer (pH 7) at 30 °C and constant stirring at 150 min^−1^. **c** AO or GAO alone or both with CAT and HRP. Reaction conditions: final reaction volume 5 mL, 1 µM AO, 8 µM GAO, 2 µM CAT and 8 µM HRP, 1 µM FAD, 10 mM HMF in 50 mM sodium phosphate buffer (pH 7) at 30 °C and constant stirring at 150 min^−1^. *HMF* 5-hydroxymethilfurfural, *HMFA* 5-hydroxymethyl-2-furoic acid, *DFF* 2,5-diformylfuran, *FFA* 5-formyl-2-furoic acid, *FDCA* 2,5-furandicarboxylic acid, *AO* alcohol oxidase from *Pichia pastoris*, *GAO* galactose oxidase from *Dactylium dendroides*, *HRP* horseradish peroxidase, *CAT* catalase from *Aspergillus niger*. The average relative error was ± 11% and was estimated based on selected repeated experiments

With the aim of achieving a full conversion of HMF to FDCA or at least increasing the productivity of an enzyme, we devised simple one-pot multi-enzyme reactions (Fig. 4a–c; values of single-enzyme reactions for AO and GAO were taken from Table 2). AO and GAO were chosen as the main enzymes because of their relatively high activity against HMF compared to other selected enzymes. Since these two enzymes produce H_2_O_2_ which in turn inhibits their activity [22, 24], CAT and HRP were added to the reaction to remove this inhibitor and in that way increase the activity of AO and GAO. Results in Fig. 4a show that adding CAT to AO increased the production of DFF from 25.5 to 97.5%, but decreased the production of FFA from 3.1 to 0%. Adding HRP to AO and CAT had a similar effect, but to a smaller degree and produced 76.6% of DFF. Figure 4b shows that adding CAT to GAO slightly increased the production of DFF, from 5.1 to 7.4% and adding HRP further increased it to 18.1%. Combining all four enzymes (Fig. 4c) resulted in production of 36.7% of DFF, but without any other products being formed.

The oxidation of HMF to FDCA is rarely performed by only one enzyme as three consecutive oxidation steps are needed and thus, an enzyme would also have to accept all of the intermediates (DFF, HMFA and FFA) as a substrate. One of the exceptions was an HMF oxidase enzyme (HMFO) from *Methylovorous* sp. which was capable of oxidizing not only HMF, but also DFF, HMFA and FFA and thus producing high yields of FDCA [13, 14]. Since no other enzyme has been shown to have such activity and wide range of substrates, other groups had to rely on finding the most favourable combinations of different enzymes to achieve a full conversion. Most often, an oxidase (alcohol or galactose) was combined with CAT, HRP or an unspecific peroxygenase [11, 20, 22–24]. The latter were added not only because of their ability to utilize/remove the inhibitory H_2_O_2_ produced by the oxidases, but also because of their activity against other substrates like FFA. Similar to previous research, our results show that unlike HMFO [13, 14], none of the selected enzymes were able to catalyse the whole conversion of HMF to FDCA but that the addition of an enzyme that utilizes H_2_O_2_ can increase the activity of another enzyme (an oxidase). The most promising enzyme combination used in our work was shown to be AO and CAT. The fact that adding HRP to the reaction lowered the production of DFF indicates that some inhibitory enzyme interactions might be taking place. This is also supported by results obtained with all four enzymes, where compared to only AO and CAT and AO, CAT and HRP, the production of DFF was lower. It has previously been shown that adding CAT and HRP to GAO increases the production of DFF from 2 to 28% [22]. There they also tested GAO with only CAT and GAO with only HRP and the results showed that while the addition of CAT and HRP increased DFF production to 23% and 46%, respectively, the combination of all three enzymes did not give the highest yield. This again points to an inhibitory enzyme interaction. On the other hand, our results show a more pronounced rise in DFF production when HRP was added to the reaction. However, we did not test GAO with only HRP and thus could not determine its effect on GAO. We would expect that this combination would yield the best results as HRP has been shown to activate GAO [11, 23, 30, 31].

## Conclusions

This study looked at the mechanism of enzymatic conversion of HMF and its oxidation products to FDCA. The activity was determined based on a single-enzyme active site and at atmospheric oxygen. Different enzymes had different substrate specificities as well as employed different HMF oxidation routes, but most had only low activity against the selected substrates. The highest yields were obtained for both oxidases. Substrate concentration affected only alcohol oxidase activity. Combining different enzymes in multi-enzyme reactions increased the conversion of HMF to DFF, and the best results were obtained with AO and CAT.

## Methods

### Materials

The enzymes used were commercially available alcohol oxidase from *Pichia pastoris* (AO), galactose oxidase from *Dactylium dendroides* (GAO), catalase from *Aspergillus niger* (CAT), laccase from *Trametes versicolor* (LAC), fungal lignin peroxidase (LPO) and horseradish peroxidase (HRP). 5-Hydroxymethylfurfural (HMF), 2,5-furandicarboxylic acid (FDCA), 5-hydroxymethyl-2-furoic acid (HMFA), 5-formyl-2-furoic acid (FFA) and 2,5-diformylfuran (DFF) were all analytical standard grade and used as standards for HPLC and/or substrates. Flavin adenine dinucleotide (FAD) was used as a cofactor for the alcohol oxidase. All the materials were obtained from Sigma Aldrich and used without further purification.

### Enzymatic reactions

All reactions were performed in 50 mM sodium phosphate buffer (pH 7) at 30 °C and constant stirring at 150 min^−1^ with 10 × 6 mm stirrers. The reactors were 15-mL amber screw cap vials with hole cap PTFE/silicone septa (Supelco). A short tube (aprox. 2 cm) was inserted through the septa to allow air passage.

The enzyme concentrations used were determined based on the number of their active sites and were 1 µM AO (8 active sites), 2 µM CAT (4 active sites) and 8 µM GAO, LAC, LPO and HRP (1 active site). The number of active sites/subunits was determined with the aid of the UniProt database [32] and manufacturers specification sheet [33], if available. For AO, flavin adenine dinucleotide (FAD) was used as a cofactor in 1, 10 or 100 µM concentration. Enzymes were mixed with 20, 10 or 5 mM HMF, DFF, FFA or HMFA, and incubated for 72 h. For controls, 10 mM HMF, DFF, FFA or HMFA were incubated in the same conditions in the absence of an enzyme or 3 mM HMF was incubated with 5 mM H_2_O_2_. Sample preparation prior to HPLC analysis was done according to previous methods [20, 23, 24] with some modifications. Two hundred microliter samples of the reaction mixtures were taken after 24, 48 and 72 h. The samples were mixed with 385 µL of MQ water and quenched with 15 µL of 1.2 M HCl to inactivate the enzyme and stop the reaction. The samples were then centrifuged through a membrane with 10-kDa cut-off (Amicon Ultra) and filtered through 0.2-µm filter (Chromafil Xtra CA-20/13, Macherey–Nagel), prior to HPLC analysis. During the reaction with AO and HMF, oxygen content was also measured (Oxi 340i/SET, WTW). The measurements were performed at the beginning of the reaction and subsequently in 24-h intervals.

Kinetic parameters were determined for reactions with selected enzymes at varying substrate concentrations: AO with HMF, GAO with DFF, LAC with HMFA and HRP with FFA. Substrate concentrations were 5, 10 and 20 mM and reaction conditions as described above. Kinetic parameters were determined based on the amount of product formed in the first 24 h of reaction. *V*_0_ was calculated based on the Michaelis–Menten equation and *V*_max_ and *K*_m_ based on Lineweaver–Burk plot.

### Analytical methods

The reaction mixture was analysed by HPLC system (Dionex Ultimate 3000, Thermo Scientific) on a Supelcogel 8H column (300 mm × 7.8 mm, Sigma Aldrich). The mobile phase consisted of 5 mM H_2_SO_4_ (pH 2) with a flow rate of 0.6 mL min^−1^. The column temperature was set at 70 °C and UV–Vis absorption wavelengths were 285 nm for HMF and DFF, 254 nm for HMFA, 272 nm for FFA and 265 nm for FDCA. Individual compounds were quantified by external calibration standards. The retention times were as follows: HMF (25.96 min), DFF (30.85 min), HMFA (18.41 min), FFA (19.21 min) and FDCA (14.77 min). Product yields (%) were calculated based on a normalized total sum of all the products and the remaining substrate.

## Data Availability

All data generated or analysed during this study are included in this published article.

## Abbreviations

HMF: 5-Hydroxymethylfurfural
FDCA: Furan-2,5-dicarboxylic acid
DFF: 2,5-Diformylfuran
FFA: Formyl-2-furoic acid
HMFA: 5-Hydroxymethyl-2-furoic acid
AO: Alcohol oxidase enzyme
GAO: Galactose oxidase enzyme
CAT: Catalase enzyme
LAC: Laccase enzyme
LPO: Lignin peroxidase enzyme
HRP: Horseradish peroxidase enzyme
FAD: Flavin adenine dinucleotide
